# High prevalence rate of left superior vena cava determined by echocardiography in patients with congenital heart disease in Saudi Arabia

**DOI:** 10.3402/ljm.v8i0.21679

**Published:** 2013-10-08

**Authors:** Ghada Shiekh Eldin, Milad El-Segaier, Mohammed Omer Galal

**Affiliations:** 1 Department of Paediatric Cardiology, PSHC, King Fahd Medical City, Riyadh, Saudi Arabia; 2 Department of Paediatric Cardiology, Skåne University Hospital, Lund, Sweden; 3 Department of Paediatric Cardiology, University of Essen, Essen, Germany

**Keywords:** left superior vena cava, Down syndrome, atrioventricular septal defect, congenital heart disease

## Abstract

**Background:**

Persistent left superior vena cava (LSVC) is one of the common anomalies of the systemic veins. Its prevalence is 0.1–0.3% in the general population and is more common with congenital heart disease (CHD). The importance of detecting persistent LSVC prior to cardiac surgery is paramount for systemic veins cannulations.

**Aim:**

The aim was to evaluate the prevalence of persistent LSVC in patients with CHD in Saudi Arabia.

**Methods:**

All patients referred to our institution had echocardiography. All complete studies were reviewed for the presence of persistent LSVC. A computerized database was created including the demographic data, CHD diagnoses, and the presence of persistent LSVC.

**Results:**

A total of 2,042 were examined with an age range of 1 day to 16 years. The complete echocardiographic studies were 1,832 (90%) of whom 738 (40%) patients had CHD. The prevalence of persistent LSVC in patients with CHD was 7.8% (OR 9.26, 95% CI 4.7–18.2, *p<*0.001). The most common cardiac defect associated with persistent LSVC was complete atrioventricular septal defect (AVSD); all patients with AVSD had Down syndrome. The total number of patients with AVSD was 41, and persistent LSVC was found in 11 (26%) of them (odds ratio 5.1, 95% CI 2.4–10.8, *p<*0.001).

**Conclusions:**

The prevalence of persistent LSVC in the current population is almost double the reported prevalence obtained using the same echocardiographic screening tool.

The persistent left superior vena cava (LSVC) was first reported by Le Chat in 1738 (1). Its prevalence in the general population was estimated to be 0.1–0.3%, and it is described as the most common venous anomaly in humans (2–4). In patients with congenital heart disease (CHD), the reported prevalence varies with the method of diagnosis. It is reported as high as 5% by echocardiography and up to 11% by invasive angiography techniques (3, 5–8). Its isolated presence rarely causes clinical symptoms and may not be diagnosed (5, 6, 9). However, in patients who need central lines or a cardiac operation, complications may occur during line insertion or cannulation for the cardiopulmonary bypass (CPB) machine (9–18).

Failure to detect its presence prior to cardiac surgery may cause some difficulties and prolong the operation time due to excessive return of blood through the right atrium and inadequate venous return of blood to the pump (19). Thus, its diagnosis and recognition in these circumstances become more important. Additionally, a description of its presence and size is more important in cases with complex CHD that will undergo univentricular pathway of repair (20, 21). It usually drains to the coronary sinus, but occasionally it may drain to the left atrium and lead to cyanosis due to venous blood flow to the systemic circulation (22).

We noticed in our clinical practice an unexpectedly higher frequency of LSVC among Saudi children with CHD. Therefore, the aim of this study was to evaluate the prevalence of persistent LSVC in patients with CHD in our patient population.

## Patients and methods

All patients referred to our center for cardiac evaluation were included in the study. Between December 2010 and December 2011, 2,042 patients were evaluated by trans-thoracic echocardiography (using Phillips iE33 system and transducers S5 Hz, S8 Hz or C12 Hz) using the standard views. The patients were referred from peripheral hospitals, emergency department, intensive neonatal care unit, pediatric intensive care unit, general pediatric units, and patients admitted for cardiac surgery or cardiac catheterization. Patients without CHD, those referred for cardiac function evaluation and patients with incomplete studies were excluded from the analysis.

Screening for persistent LSVC is part of our complete echocardiographic evaluation. The detection of persistent LSVC was established using three different echocardiographic views as described elsewhere (8). The LSVC is visualized from the suprasternal notch or from high parasternal view, and its course is followed up to its entrance to the coronary sinus or left atrium. From a parasternal long axis view, an enlarged coronary sinus is also viewed. Evaluating and searching for the presence of an innominate vein is also done. All echocardiographic studies were reviewed offline for the presence of persistent LSVC. A computerized database was created, including demographic data, diagnosis of CHD, presence of persistent LSVC, and associated diseases or syndromes.

The institutional review board (IRB) at King Fahad Medical City approved the study. Because the study was retrospective, the IRB waived the need for patient consent.

## Statistical analysis

The collected data were analyzed using SPSS statistical software (SPSS Inc., Version 14). The continuous variables are presented as mean and median and categorical variables as number of observed patients (percentage). A *p*-value *<*0.05 was used as the level of significance. Odd ratios were calculated at a 95 confidence interval (CI) for the presence of persistent LSVC or absence of CHD, and for an association to a specific diagnosis.

## Results

We evaluated 2,042 patients ranging in age from 1 day to 16 years (mean age of 29 months and median age of 10 months). Complete echocardiographic studies were carried out for 1,832 (90%) patients; 738 patients (40%) were diagnosed with CHD. Patients who were referred for anatomical cardiac evaluation and found to be normal (*n=*666), and those who were referred for myocardial function assessment (*n=*638) were excluded from the final analysis.

Persistent LSVC was detected in 58 out of 738 patients with CHD, giving a prevalence of persistent LSVC of 7.8% (OR 9.26, 95% CI 4.7–18.2, *p<*0.001). The percentage of occurrence of persistent LSVC with different cardiac lesions is presented in Table 1.

**Table 1:** The association of the different congenital heart disease with left superior vena cava

Diagnosis	No. of patients with CHD	No. of patients with LSVC (%)
VSD, VSD with DORV	151	7 (4.6)
ASD	255	10 (3.9)
AVSD	41	11 (26.3)
PDA	105	4 (3.8)
PA, PS, DCRV and Ebstien	95	9 (9.4)
TOF, DORV with TOF	21	5 (23.8)
TGA, DORV with TGA	10	1 (10)
Co. A	18	5 (27)
AS, BAV, subaortic membrane	30	1 (3.3)
MA, HLVS	2	2 (100)
Truncus arteriosus	2	0 (0)
TAPVD	2	0 (0)
Single ventricle (DILV, DORV hetrotaxia)	3	1 (33)
Complex	2	1 (50)
Interrupted IVC (isolated)	1	1 (100)

CHD=congenital heart disease, LSVC=left superior vena cava, VSD=ventricular septal defect, DORV=double outlet right ventricle, ASD=atrial septal defect, AVSD=atrioventricular septal defect, PDA=patent ductus arteriosus, PA=pulmonary atresia, PS=pulmonary stenosis, DCRV=double chambered right ventricle, TOF=tetralogy of Fallot, TGA=transposition of the great arteries, Co. A=coarctation of the aorta, AS=aortic stenosis, BAV=bicuspid aortic valve, MA=mitral atresia, HLVS=hypoplastic left ventricle syndrome, TAPVD=total anomalous pulmonary venous drainage, DILV=double inlet left ventricle, IVC=inferior vena cava.

Out of 738 patients with CHD, 41 patients (19%) had complete atrioventricular septal defect (AVSD) and Down syndrome. Persistent LSVC was detected in 11 (26%) of this group of patients (odds ratio 5.1, 95% CI 2.4 to 10.8, *p<*0.001).

Out of 58 patients with persistent LSVC, 28 patients had cardiac surgery. In 89% of these patients, persistent LSVC was detected by echocardiography before surgery. All patients who underwent cardiac catheterization and confirmed to have persistent LSVC had already been diagnosed by echocardiography before the procedure.

Two patients who had persistent LSVC also had VACTERL syndrome (vertebral defects, anal atresia, cardiac malformations, tracheoesophageal fistula with esophageal atresia, radial and renal dysplasia, and limb anomalies). Another patient had CHARGE association (coloboma, heart defects, atresia of choanae, mental retardation, genital and ear anomalies) as well as persistent LSVC.

In all cases, persistent LSVC drained to the coronary sinus except for one patient in whom it drained to the left atrium with no significant effect on saturation. Right SVC was found in all cases with persistent LSVC, and a small innominate vein was detected in three cases.

## Discussion

The reported prevalence of persistent LSVC in CHD patients varies widely according to the diagnostic tool utilized. Buirski et al. reported a prevalence rate of 11% by using invasive catheterization and angiography for diagnosis (6). Huhta et al. reported a lower rate (between 1.3 and 5%) by retrieving all cases of persistent LSVC diagnosed by angiography, echocardiography, or autopsy for detection (23). What is noteworthy is that the prevalence we detected is almost double the prevalence reported by Postema et al., who used the same echocardiographic screening tool (8). Ethnic background might be one factor causing the above-mentioned discrepancy.

In the current study, all cases with AVSD had Down syndrome (*n=*41) and 11 (26%) had persistent LSVC. This shows a strong association between persistent LSVC and both AVSD and Down syndrome. This figure is different from what was reported by Postema et al., who found an association between persistent LSVC and AVSD but not with Down syndrome (8). Buirski et al. also reported that 19% of patients with AVSD had persistent LSVC (6).

There was a similar high prevalence, in the current study, of persistent LSVC in patients with tetralogy of Fallot and coarctation of the aorta when compared to other studies (6, 8). This can be explained by the fact that the patient sample in the current study with these lesions was small. The prevalence of persistent LSVC was low in cases of transposition of the great arteries; this is similar to what is reported in the literature (23).

The presence of persistent LSVC was diagnosed using echocardiography as the only diagnostic tool in all of the patients. This was confirmed later in some of them during surgery and catheterization. The reported sensitivity and specificity for diagnosing persistent LSVC by echocardiography were 68–96% and 100%, respectively. The detection rate for persistent LSVC is higher when the operator actively searches for its presence and has a high level of suspicion. This may partly explain the higher prevalence in our study compared to the previous one.

The association of persistent LSVC with CHARGE, VACTREL, esophageal atresia, and Turner syndrome was high as reported in one study (8). Therefore, in patients with these syndromes one should search for persistent LSVC before intravenous line insertion (8). When persistent LSVC is present, it usually drains into the right atrium via coronary sinus in 92% of the cases (24). In our study it does so in all cases but one, in which it drained to the left atrium and had no significant effect on the saturation. The presence of persistent LSVC has particular hemodynamic and clinical significance when it drains to the left atrium, as these patients will have severe cyanosis and an increased risk of paradoxical cerebral thromboembolism, which may cause cerebral sequelae, though sometimes it does not have any clinical significance, as in our case. Absent right SVC was reported in 20% of cases who had persistent LSVC (24). In our patients there was no absent right SVC.

Discovering a previously undiagnosed persistent LSVC during CBP may present some inconvenience for both perfusionist and the surgeon, especially during a minimally invasive approach (25). Failure to describe the presence of persistent LSVC prior to cardiac surgery will cause an excessive return of blood through the right atrium during CPB and inadequate venous return to the pump. This is important in cases like tetralogy of Fallot, truncus arteriosus and pulmonary atresia, where there is an increase in systemic venous pressure above the left atrial pressure (6). Intensive care clinicians should be aware of the presence of persistent LSVC in order not to insert the catheter outside the venous circulation and to avoid complications like perfusion, shock, cardiac arrest, cardiac tamponade and thrombosis (24).

The study of the variant types of venous system is essential before CPB, such as Glenn anastomosis (bidirectional cavo-pulmonary connection) or total cavo-pulmonary connection. Additionally, the description of the presence and size of persistent LSVC is important when bilateral bidirectional Glenn is planned, when the surgeon needs to anastomose the persistent LSVC to the left pulmonary artery and the RSVC to the right pulmonary artery. This issue is more significant in patients with hetrotaxia, where the incidence of persistent LSVC is high. Furthermore, drainage of persistent LSVC to coronary sinus will result in its dilatation. Thus, in mitral valve replacement, the presence of dilated coronary sinus may present the surgeon with some difficulties.

## Conclusions

Persistent LSVC in Saudi children with CHD obtained by echocardiography is more prevalent than reported elsewhere using the same screening tool and is commonly associated not only with AVSD but also with Down syndrome. Echocardiography could be sensitive tool for active detection of persistent LSVC.
